# Saturating light and not increased carbon dioxide under ocean acidification drives photosynthesis and growth in *Ulva rigida* (Chlorophyta)

**DOI:** 10.1002/ece3.1382

**Published:** 2015-01-25

**Authors:** Ralf Rautenberger, Pamela A Fernández, Martina Strittmatter, Svenja Heesch, Christopher E Cornwall, Catriona L Hurd, Michael Y Roleda

**Affiliations:** 1 Department of Botany, University of OtagoP.O. Box 56, Dunedin, 9054, New Zealand; 2 The Scottish Association for Marine Science, Scottish Marine InstituteOban, Argyll, PA37 1QA, Scotland; 3 Irish Seaweed Research Group, Ryan Institute for Environmental, Marine and Energy Research, National University of IrelandGalway (NUIG), University Road, Galway, Ireland; 4 Institute for Marine and Antarctic Studies, University of TasmaniaHobart, Tasmania, 7001, Australia; 5 Bioforsk Norwegian Institute for Agricultural and Environmental ResearchKudalsveien 6, 8049, Bodø, Norway

**Keywords:** Bicarbonate, C:N ratio, carbon physiology, carbon-concentrating mechanism, carbonic anhydrase, chlorophyll fluorescence, *F*_v_/*F*_m_, pigments, seaweed, stable carbon isotope

## Abstract

Carbon physiology of a genetically identified *Ulva rigida* was investigated under different CO_2(aq)_ and light levels. The study was designed to answer whether (1) light or exogenous inorganic carbon (Ci) pool is driving growth; and (2) elevated CO_2(aq)_ concentration under ocean acidification (OA) will downregulate CA_ext_-mediated 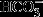 dehydration and alter the stable carbon isotope (*δ*^13^C) signatures toward more CO_2_ use to support higher growth rate. At pH_T_ 9.0 where CO_2(aq)_ is <1 *μ*mol L^−1^, inhibition of the known 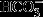 use mechanisms, that is, direct 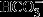 uptake through the AE port and CA_ext_-mediated 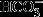 dehydration decreased net photosynthesis (NPS) by only 56–83%, leaving the carbon uptake mechanism for the remaining 17–44% of the NPS unaccounted. An in silico search for carbon-concentrating mechanism elements in expressed sequence tag libraries of *Ulva* found putative light-dependent 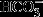 transporters to which the remaining NPS can be attributed. The shift in *δ*^13^C signatures from –22‰ toward –10‰ under saturating light but not under elevated CO_2(aq)_ suggest preference and substantial 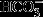 use to support photosynthesis and growth. *U. rigida* is Ci saturated, and growth was primarily controlled by light. Therefore, increased levels of CO_2(aq)_ predicted for the future will not, in isolation, stimulate *Ulva* blooms.

## Introduction

The world's oceans are a sink for CO_2_ that has been released through anthropogenic processes since the industrial revolution (∽1850). This process buffers climate change in the terrestrial system, but perturbs the seawater carbonate system, and is reducing the pH of the surface ocean, termed ocean acidification (OA) (Takahashi et al. 2014). In the next century, atmospheric CO_2_ concentrations are projected to increase from 395.93 ppm (October 2014; www.CO2now.org) to a maximum of 1142 ppm in 2100 (IPCC 2013): the 192% increase will consequently reduce global surface ocean pH by 0.4 units (Meehl et al. 2007). OA may have a profound impact on organismal carbon physiology, trophic dynamics, biogeochemical cycles, and ecosystem functions and services (Gattuso and Hansson 2011; Roleda and Hurd 2012; Koch et al. 2013).

Seaweeds are macroscopic, multicellular marine algae. They are foundation species and provide energy to higher trophic levels. They belong to three distinct evolutionary groups: green (Chlorophyta), brown (Ochrophyta), and red (Rhodophyta) seaweeds. Most seaweeds are able to take up both CO_2_ and 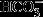 from the surrounding seawater as inorganic carbon (Ci) sources for photosynthesis. CO_2_ can enter seaweed cells through the plasma membranes by passive diffusion. However, this process is very slow in water and dissolved carbon dioxide (CO_2(aq)_) makes up less than 1% of the total Ci. On the other hand, 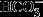 is the most abundant ionic form of Ci (92%) but requires specific carriers for transport across membranes (Björk et al. 1992; Sharkia et al. 1994).

To overcome the low availability of CO_2(aq)_ to supply the ribulose-1,5-bisphosphate carboxylase/oxygenase (RuBisCO) for photosynthetic carbon fixation, most seaweeds have developed strategies of utilizing 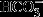. These strategies include as follows: (1) extracellular carbonic anhydrase (CA_ext_)-mediated 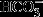 dehydration and subsequent passive diffusion of CO_2_ into the cell (Björk et al. 1992). (2) Direct 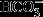 uptake by the anion-exchange protein AE1, similar to that present in mammalian red blood cells (Drechsler and Beer 1991; Drechsler et al. 1993; Sharkia et al. 1994). The internal inorganic carbon (Ci) pool is stored primarily as 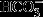; consequently, this requires intracellular carbonic anhydrase (CA_int_)-mediated 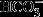 dehydration to supply CO_2_ to RuBisCO for photosynthetic carbon fixation (Fernández et al. 2014). For the seaweed genus *Ulva*, that is the focus of this study, the relative contribution of CA_ext_-mediated 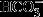 utilization versus direct 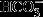 uptake to supply CO_2_ for photosynthesis varies substantially among different species where species are identified based on traditional taxonomic classification (Larsson and Axelsson 1999). (3) An indirect 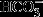 use operates in conjunction with metabolically mediated proton (H^+^) flux where H^+^ extrusion creates a local acidified zone within the diffusion boundary layer at the seaweed surface, thereby stimulating a pH-dependent conversion of 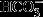 to CO_2_ (Björk et al. 1992; Beer et al. 2008). Such uncatalyzed or spontaneous dehydration of 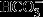 can, however, be slow and may be insufficient to support high photosynthetic and growth rates (Cook et al. 1986).

*Ulva* is a globally ubiquitous seaweed, well known for causing massive macroalgal blooms that can have negative environmental and economic impacts (McGlathery 2001; Sun et al. 2008; Pang et al. 2010). The drivers of *Ulva* primary production and growth are variously attributed to light (Aldridge and Trimmer 2009), inorganic nutrients (Coutinho and Zingmark 1993), CO_2_ (Xu and Gao 2012), and their interactions. Under a saturating light (200–500 *μ*mol photons m^−2^ s^−1^) growth of *Ulva curvata* can become limited by the supply of inorganic nitrogen (Coutinho and Zingmark 1993). When grown under a subsaturating light (100 *μ*mol photons m^−2^ s^−1^) (but unspecified nutrient concentration), the growth of *Ulva prolifera* cultivated from spores was reported to increase by ∽20% d^−1^ when CO_2_ was increased by 156% (Xu and Gao 2012). Whether or not a saturating light will further enhance the growth of *Ulva* under elevated CO_2_ is unknown.

There are many studies showing that 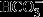 is the primary source of Ci for *Ulva* species (Drechsler and Beer 1991; Björk et al. 1992, 1993; Drechsler et al. 1993; Sharkia et al. 1994). Therefore, it is unlikely that the predicted increase in CO_2(aq)_ due to OA will have an effect on the rates of photosynthesis and growth in *Ulva* (e.g. Xu and Gao 2012). Nevertheless, researchers continue to pursue this line of research (e.g. Pajusalu et al. 2013; Olischläger et al. 2013), suggesting that increased levels of CO_2(aq)_ will cause increased growth rate in *Ulva*. Here, we demonstrate that light, and not Ci, is the main driver for growth of *Ulva* and suggest that increased levels of CO_2(aq)_ predicted for the future will not, in isolation, stimulate photosynthesis and growth of *Ulva*.

This study is important and timely because it questions the validity of the current dogma that increasing CO_2_ will promote (harmful) algal blooms and drive ecosystem “winners and losers”. There is currently a very limited understanding of algal carbon physiology on an organismal level (e.g. Kübler et al. 1999) and on the environmental drivers of basic physiological mechanisms (e.g. Raven 1991) such as photosynthesis and growth. Moreover, the combined physiological and molecular approaches to investigate key driver for *Ulva* growth confers novelty in this work.

The photosynthetic carbon physiology and growth of a genetically identified *Ulva* species under different CO_2(aq)_ and light levels were investigated. We hypothesized that (1) under current concentration of CO_2(aq)_, exogenous Ci is saturating for photosynthesis and growth of *Ulva rigida*; (2) *U. rigida* has several putative 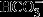 use mechanisms other than the known inhibitor-sensitive CA_ext_-catalyzed dehydration of 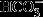 and direct uptake of 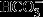 through the anion-exchange (AE) port; and (3) elevated concentrations of CO_2(aq)_ predicted for the future will not affect *U. rigida* growth. The experiment was designed to answer whether (1) light or exogenous Ci pool is limiting *U. rigida* growth; (2) elevated Ci (CO_2_ and 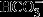) under OA will support a higher growth rate; (3) CA_ext_-mediated 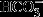 dehydration is downregulated when *Ulva* is grown under a high CO_2(aq)_ concentration; and (4) the hypothetical shift to more CO_2_ use under OA will alter the stable carbon isotope (*δ*^13^C) signature (*cf* Maberly et al. 1992; Raven et al. 2002; Giordano et al. 2005) and the higher available Ci under OA relative to constant nutrient supply will increase the molar carbon to nitrogen (C:N) ratio (*cf* van de Waal et al. 2010).

## Materials and Methods

### Algal material and stock culture conditions

**Figure 1:**
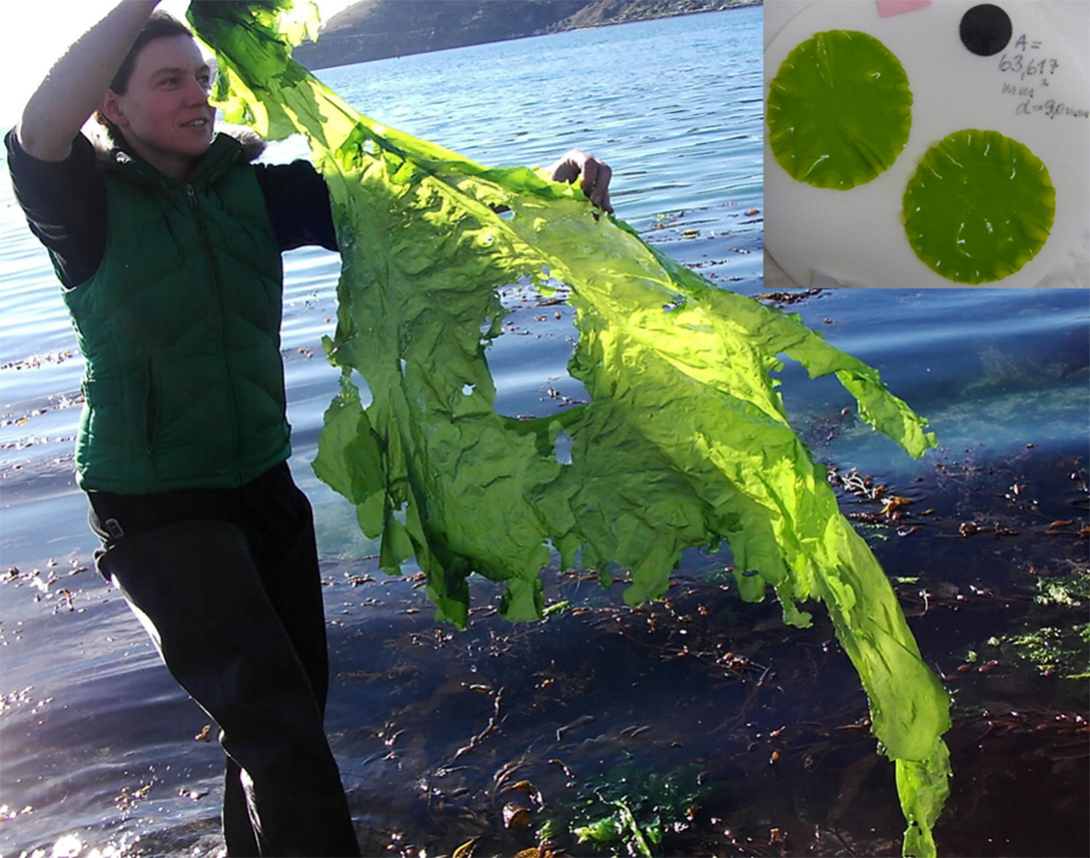
Sheet-forming *Ulva* from the subtidal flat at the entrance of Otago Harbour, southern New Zealand. Inset: discs of genetically identified *Ulva rigida* clones used in the experiment.

Sheet-forming *Ulva* thalli (Fig.1) were collected on 5 October 2011 from the subtidal at the entrance (3 m depth) of Otago Harbour, southern New Zealand (Aramoana, 45.8°S, 170.7°E) and transported in a cooled container to the laboratory. Several algal discs (Fig.1 inset) were excised from one individual and cultivated in 5-L glass vessels with nutrient-enriched seawater, formulated using the ESNW recipe (Harrison et al. 1980; Berges et al. 2001), which was used throughout the experiment. To avoid significant pH drop, the ESNW was prepared with one-third of the full strength concentrations of phosphate, iron-EDTA (pH <6.0), trace metals II (pH <6.0) and vitamins stock solutions and 5 *μ*mol L^−1^ of NaNO_3_-N; the final seawater pH was pH_T_ 7.97, ∽0.08 pH unit lower than ambient seawater. The *Ulva* clones were grown under photosynthetic photon fluence rate (PPFR) of 50 *μ*mol photons m^−2^ s^−1^ over the waveband 400–700 nm (Philips TLD 18W/840 Cool White; Philips, Amsterdam, The Netherlands) and 12 h: 12 h, light:dark cycle at 13°C in a Phytotron Climate Simulator cabinet (Contherm Scientific Ltd, Lower Hutt, New Zealand). Clones were grown below the saturating irradiance of 79 ± 9 *μ*mol photons m^−2^ s^−1^ (R. Rautenberger, unpubl. data) to avoid rapid growth, nutrient depletion, and swarmer release. After 8 months clonal propagation, algal discs (3 cm^2^, 25 mg FW) were used in the experiments below.

Two specimens grown from the clonal cultures were prepared as herbarium vouchers and deposited in the herbarium of the University of Otago (OTA; Thiers 2014) under the accession numbers OTA63969 and OTA63970. Material of the latter specimen was prepared for genetic identification by excising a disc from the freshly harvested thallus and drying it in silica gel.

### Genetic identification and life history of the cultured strain

The *Ulva* strain (SBDN 247) was genetically identified using the methods in Heesch et al. (2009), except for the following differences: DNA was extracted and PCR products were purified using commercial kits (NucleoSpin® Plant II, Macherey-Nagel, Düren, Germany, and PureLink® PCR Purification Kit, Invitrogen, Germany, respectively). Sequence alignments of the large subunit of the plastid-encoded RuBisCO gene region (*rbc*L) were analyzed under the maximum-likelihood (ML) criterion using the default settings in RAxML v.7.2.2 (Stamatakis 2006). Our *rbc*L sequence (European Nucleotide Archive [ENA] accession no. LK022428) was found to be 100% identical to *rbc*L sequences from *U. rigida* samples collected in New Zealand (e.g. GenBank accession number EF110302; Heesch et al. 2009), Europe (EU484408; Loughnane et al. 2008) and Chile (AY422564: Hayden and Waaland 2004). The phylogenetic analysis placed our strain in a well-supported clade with the above sequences (data not shown), confirming its identification as *Ulva rigida* C.Agardh.

Swarmers released from clonal tissue were positively phototactic indicating that the cultivated strain was a gametophyte, that is, the haploid generation (Guiry and Guiry 2014). Cultivation of the released gametes failed to develop parthenogenetically, suggesting that swarmers were most likely male gametes, which, in contrast to female swarmers, have lower capacity to germinate without fertilization (Koeman and van den Hoek 1981).

### Carbon physiology: inhibition of known bicarbonate-use mechanisms

Acetazolamide (AZ, CAS number 59-66-5) and 4,4′-diisothiocyanatostilbene-2,2′-disulfonate (DIDS, CAS number 207233-90-7) are inhibitors with high specificity for blocking the catalyzed external dehydration of 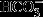 and the direct 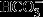 uptake through the anion-exchange (AE) port, respectively (Björk et al. 1992; Axelsson et al. 1995, 1999; Axelsson et al. 2000; Herfort et al. 2002; Suffrian et al. 2011). Both mechanisms operate independently: the addition of these inhibitors in the absence of other 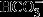 uptake mechanisms can result in an almost complete inhibition of net photosynthesis (Axelsson et al. 1995; Larsson and Axelsson 1999; Fernández et al. 2014). A concentration of 300 *μ*mol L^−1^ DIDS to inhibit the direct HCO_3_^−^ uptake and 100 *μ*mol L^−1^ AZ to inhibit CA_ext_ were selected based on the dose response curves of Herfort et al. (2002), and the standard utilization of these concentrations across studies on the Ci-use mechanisms of micro- and macroalgal species (e.g. Björk et al. 1992; Axelsson et al. 1995, 1999; 2000; Young et al. 2001; Herfort et al. 2002; Suffrian et al. 2011; van Hille et al. 2014). The 10 mmol L^−1^ stock solution of AZ (≥99%, Sigma-Aldrich, St. Louis, MO, USA) was prepared by dissolving the powder in a basic medium (10 mmol L^−1^ NaOH in MilliQ, 18.3 MΩ cm) while the 30 mmol L^−1^ stock solution of DIDS (≥80% elemental analysis, Sigma-Aldrich) was prepared by dissolving the powder in MilliQ. The stock solutions were freshly prepared and kept at 4°C and dark.

Algal discs were acclimated for 2 days under pH_T_ 9.0 where Ci is mainly available as 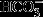 (700 *μ*mol L^−1^) with minimal CO_2(aq)_ (<1 *μ*mol L^−1^). Seawater was adjusted using equal amounts of 0.2 mol L^−1^ NaOH and 0.2 mol L^−1^ NaHCO_3_ (Roleda et al. 2012a). After 2 days, inhibition of photosynthetic O_2_ evolution (*n* = 3) under the same pH_T_ = 9.0 was measured inside a 154-mL transparent acrylic glass chamber equipped with an optode, that is, FOXY–R fiber optic oxygen sensor coupled to the USB–2000 spectrophotometer (Ocean Optics, Dunedin, FL, USA) and the PC interface. The seawater was continuously stirred (650 rpm) to create a homogenous O_2_ profile. To avoid photorespiration due to high O_2_ concentrations and temperature, the seawater was initially adjusted to 100 ± 20 *μ*mol O_2_ L^−1^ using N_2_ gas and kept constant at 12 ± 1°C, respectively. Net oxygen evolution of individual *U. rigida* discs was recorded (OOISensor 1.0 software; Ocean Optics Inc., FL, USA) at a PPFR of 250 *μ*mol photons m^−2^ s^−1^ (400–700 nm). Oxygen concentration inside the chamber was recorded for 20 min before the inhibitors were added and then for a further 2 × 15 min following the sequential addition of the two inhibitors (e.g. DIDS-AZ and AZ-DIDS; *n* = 3). The oxygen concentration was measured continuously for a total of 50 min per sample. The rate of oxygen evolution was calculated using a linear regression for every stepwise incubation period, that is, the whole incubation time (20 min) before application of the inhibitors, and after every application of each inhibitor (2 × 15 min). The oxygen concentration (*μ*mol O_2_ L^−1^) was measured after Millero and Poisson (1981) and García and Gordon (1992), and corresponding % inhibition of net photosynthetic (NPS) rate was calculated.

### In silico detection of carbon-concentrating mechanisms elements in expressed sequence tag libraries of *Ulva*

To test our hypothesis on *Ulva* having different Ci uptake mechanisms other than the known inhibitor-sensitive CA_ext_-catalyzed dehydration of 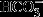 and direct uptake of 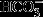 through the AE port, we searched one expressed sequence tag (EST) library of *U. prolifera* (Jia et al. 2011) publicly available in the dbEST database (Boguski et al. 1993) for putative carbon-concentrating mechanism (CCM) elements. A number of described CCM proteins of the unicellular green alga *Chlamydomonas reinhardtii* were used in a tblastn search against the *U. prolifera* EST data set and included the following protein sequences: alpha carbonic anhydrases (CAH1 [accession number BAA14232], CAH3 [EDP00852.1]), beta carbonic anhydrases (CAH6 [AAR82947.1], CAH8 [ABS87675.1]), gamma carbonic anhydrase (CAG2 [XP_001701594]), nuclear transcriptional regulators of CCM elements (CIA5 [AAG37909.1], LCR1 [BAD13492.1]), low-CO_2_-inducible chloroplast membrane Ci candidate transporter (LCIA [XP_001703387.1]), low-CO_2_-inducible proteins to recapture leaking CO_2_ (LCIB [EDP04243.1], LCIC [BAD16683.1]), chloroplast carrier protein 1 (CCP1 [EDP04147.1]), high light-activated 3 (HLA3) ATP-binding cassette (ABC) transporter (HLA3 [XP_001700040.1]) and chloroplast proton extrusion protein (CemA [XP_001696592]). Retrieved sequences were further subjected to a reciprocal blastx search against the National Center for Biotechnology Information (NCBI) Genbank nonredundant (nr) database. Furthermore, a VecScreen was run on retrieved sequences to check for vector contamination.

### Interactive light × CO_2_ experimental set-up

Six discs of *U. rigida* clones (each 3 cm^2^) were contained in each 24 × 650 mL Perspex flow-through culture vessels. Specimen was pre-incubated under pH_T_ 7.96 and nutrient replete condition (11.6 ± 0.7 *μ*mol L^−1^ NO_3_-N, 9.1 ± 0.4 *μ*mol L^−1^ PO_4_-P and 0.6 ± 0.1 *μ*mol L^−1^ NH_4_-N; ± SD, *n* = 3), exposed to PPFR of 30 *μ*mol photons m^−2^ s^−1^ (400–700 nm, 12 h: 12 h, light:dark) in-side a temperature-controlled walk-in culture chamber set to 13 ± 1°C. A preliminary rapid ETR-*E* curve measurement calculated a saturating irradiance *E*_*k*_ = 79 ± 9 *μ*mol photons m^−2^ s^−1^, and no photo-inhibition of the maximum electron transport rate (ETR_max_) was observed up to a maximum PPFR of 600 *μ*mol photons m^−2^ s^−1^ (R. Rautenberger, unpubl. data). Accordingly, two light treatments (PPFR of 400–700 nm) were set as follows: a limiting light (LL) of 31 ± 9 *μ*mol photons m^−2^ s^−1^ and a saturating light (SL) of 274 ± 18 *μ*mol photons m^−2^ s^−1^. Philips HPI- T 400 W quartz metal halide lamps (Philips) provided the experimental light and the PPFR measured using a 4*π* quantum sensor (QSL-2101, Biospherical Instruments Inc., San Diego, CA, USA). The LL treatment was achieved using layers of neutral density black screen cover.

The response of *U*. *rigida* to a shift in *p*CO_2_ was investigated under two treatments slightly higher than today's *p*CO_2_ (471 *μ*atm) and that predicted for year 2100 (1224 *μ*atm). The 160% higher CO_2(aq)_ between the low and high *p*CO_2_ treatments is slightly lower than the 192% increase projected in 2100 (Meehl et al. 2007; IPCC 2013). After three days pre-incubation described above, each culture vessel was randomly assigned to different *p*CO_2_/pH_T_ (high CO_2_/low pH_T_ 7.59 = HC; low CO_2_/high pH_T_ 7.97 = LC) and light (LL; SL) treatments under higher nitrate concentration (90 *μ*mol L^−1^ NO_3_-N). The two factorial experiment resulted in four treatment combinations of light and *p*CO_2_: HC/LL, HC/SL, LC/LL and LC/SL (*n* = 6).

Manipulation of the seawater carbonate system was achieved using a modified version of the automated pH-controlled culture system described by McGraw et al. (2010). Briefly, target pH (pH_T_) levels were achieved by adding HCl and NaHCO_3_ (Hurd et al. 2009; Riebesell et al. 2010) to the nutrient-enriched seawater and measured spectrophotometrically to an accuracy of 0.03 pH units. Thereafter, the pH-adjusted seawater automatically supplied fresh medium to the respective randomly assigned culture vessel. Seawater in each of the 24 vessels was refreshed every 4.4 h (=4× a day change in medium). Within the 4.4-h incubation period, a maximum 0.18 units increase in pH due to photosynthesis was estimated (Cornwall et al. 2012); thereby persistently exposing the algae to a specific range of the pH treatment. Each culture vessel was provided with water movement using magnetic bars stirred at 650 rpm. Five hundred milliliter of the acid–base manipulated seawater corresponding to pH_T_ 7.59 and 7.97 was collected and fixed with mercuric chloride. Total alkalinity (A_T_) of samples was measured using the closed-cell titration method described by Dickson et al. (2007). A_T_, pH, salinity, and temperature were used to calculate carbonate chemistry parameters using the program SWCO2 (Hunter 2007). The seawater carbonate chemistry is presented in Table1.

**Table 1:** Summary of carbonate chemistry of seawater used in the experiment

Parameter (unit)	Ambient CO_2_ LC	Elevated CO_2_ HC
A_T_ (*μ*mol kg^−1^)	2197.44 ± 11.99	2207.55 ± 15.97
DIC (*μ*mol kg^−1^)	2031.41 ± 11.23	2170.76 ± 15.74
H_2_CO_3_ (*μ*mol kg^−1^)	18.49 ± 0.11	48.02 ± 0.51
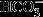 (*μ*mol kg^−1^)	1890.43 ± 10.37	2066.36 ± 14.97
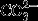 (*μ*mol kg^−1^)	122.48 ± 0.88	56.38 ± 0.66
*p*CO_2_ (*μ*atm)	471.38 ± 2.87	1223.91 ± 13.02
pH_T_	7.965 ± 0.002	7.590 ± 0.004

Carbonate parameters were calculated from total alkalinity (A_T_) and pH_T_ measurements corresponding to each *p*CO_2_ treatment at 13 ± 1°C. Seawater was filtered and nutrient-enriched (ENSW formula), salinity 34 psu. Data are means ± standard deviations (*n* = 12).

After 7 days, samples were analyzed for growth, photosynthetic efficiency, pigments, stable isotope signatures, and internal and external carbonic anhydrase (CA) activity. For biochemical analyses, samples were stored either at –20°C (pigment) or –80°C (CA) until analysis.

### Growth rate

Algal discs (Fig.1 inset) from each culture vessel were blotted dry and photographed by a 14-megapixels resolution digital camera (Lumix DMC-FT10; Panasonic, Osaka, Japan). Algal surface area (cm^2^) was analyzed by comparing the pixel density of algal discs to a known reference area using the software ImageJ version 1.47b (National Institutes of Health, Bethesda, MD, USA, http://rsb.info.nih.gov/ij/). Relative growth rate (RGR; % d^−1^) was calculated from the surface area at the start and end of the experiment after Lüning (1990).

### Chlorophyll *a* fluorescence

The Chl*a* fluorescence of each algal disc was measured submerged in their respective pH treatment using a PAM-2000 fluorometer (Walz GmbH, Effeltrich, Germany) following the protocol of Rautenberger et al. (2009) at 13 ± 1°C. After 5-min dark incubation, basic (F_0_) and maximum fluorescence (F_m_) was measured and *F*_v_/*F*_m_ was calculated (Schreiber et al. 1995). Electron transport rate (ETR) vs. irradiance (*E*) curves (ETR–*E* curves) were recorded from discs exposed to incrementally increasing (every 30 s) actinic light (AL) intensities (7–500 *μ*mol photons m^−2^ s^−1^; 400–700 nm). All saturation pulses were set to >9000 *μ*mol photons m^−2^ s^−1^ and 0.8 s. The ETRs through the photosystem II (PSII) were calculated by multiplying the intensity of incident AL, the proportion of incident AL intensity that was absorbed by the measured disc, the fraction of absorbed AL which is most probably received by PSII (0.5) and the PSII operating efficiency (Baker 2008). Maximum electron transport rates (ETR_max_), the initial slopes of ETR–*E* curves, called *α*, and the light saturation points of ETRs (i.e., *E*_*k*_) were estimated from ETRs plotted against the incident AL intensity and calculated according to the model of Jassby and Platt (1976) using the software R version 2.15 (The R Foundation for Statistical Computing, http://www.R-project.org).

### Pigments

Frozen algal discs were extracted in 3 mL of 100% N-N-dimethylformamide (DMF; BDH Laboratory Supplies, UK) at 4°C in the dark for 42 h. Chl*a* and Chl*b* were analyzed photometrically at 663.8 and 646.8 nm (Ultrospec 3000; Pharmacia Biotech, Cambridge, UK) with 100% DMF as reference at 20°C. Readings at 750.0 nm were used as a correction for scattering light. Chl*a* and Chl*b* contents were calculated after Porra et al. (1989) and normalized to algal biomass (*μ*g mg^−1^ FW).

### Extracellular and intracellular carbonic anhydrase activity

Carbonic anhydrase activity was measured according to the pH drift method of Wilbur and Anderson (1948) and Haglund et al. (1992a) using 50 mmol L^−1^ Tris HCl buffer (adjusted to pH 8.5, 4°C), 2 mmol L^−1^ dithiothreitol (DTT), 15 mmol L^−1^ ascorbic acid and 5 mmol L^−1^ EDTA (disodium salt). The external carbonic anhydrase (CA_ext_) activity was analyzed from frozen algal discs (60 ± 20 mg FW). Each disc was placed in a 20-mL scintillation vial containing 10 ml of the extraction buffer and equipped with a micro stirrer bar (10 × 3 mm) for the enzymatic reaction. The glass vial was placed inside an ice-containing 100-mL plastic container to maintain the temperature at 0–2°C, sitting on top of a magnetic stirrer to stir the solution. Temperature and pH were simultaneously measured using a ROSS electrode (Orion 8107BNUMD) coupled to Orion 3–Stars Plus pH Benchtop meter (Orion, Thermo Fisher Scientific, Waltham, MA, USA). When pH stabilized at 8.3, 5 mL of ice-cold CO_2_-saturated water was added. The time (sec) taken for the pH to drop by 0.4 units, in the pH interval of 8.3–8.1, was recorded. The internal carbonic anhydrase (CA_int_) activity was subsequently measured from the same algal disc (Fernández et al. 2014). The algal disc was ground to fine powder in liquid N_2_-frozen mortar and pestle. The ground tissue (60 ± 20 mg) was analyzed following the protocol described above. Relative enzyme activity (REA) was computed after the formula of Haglund et al. (1992b).

### Carbon to nitrogen ratios and stable carbon isotope signatures

Molar C:N ratios and *δ*^13^C signatures (‰) were analyzed after algal discs were dried at 60 ± 1°C for 24 h. Discs were ground in a mortar and pestle to a fine powder and combusted in a CE NA1500 Elemental Analyzer (Carlo-Erba Instruments Ltd, Hindley Green, Wigan, UK) interfaced to an IRMS 20-20 continuous flow mass spectrometer (Europa Scientific Ltd, Crewe, UK). Corrections for drift were made automatically every five samples from an EDTA standard with a known isotope ratio.

### Data analyses

Means ± standard deviations (SDs) were calculated from five (*n* = 5) or six (*n* = 6) measurements per treatment. Statistical analyses were performed using JMP Pro 10.0 (SAS Institute Inc., Cary, NC, USA) and R versions 2.7 and 2.15 (The R Foundation for Statistical Computing, http://www.R-project.org). Two-way ANOVA and *t*-test were used to identify statistical differences of the means within and between treatments. Tukey's honestly significant difference (HSD, *P* < 0.05) test was used as a post hoc test.

## Results

### Carbon physiology and in silico detection of putative CCM from EST libraries

**Figure 2:**
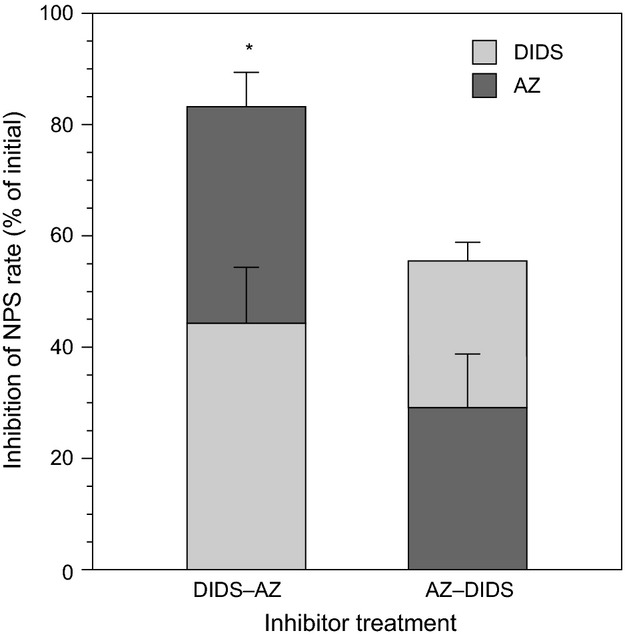
Photosynthetic inhibition after sequential blocking of the anion-exchange (AE) port followed by the inhibition of external carbonic anhydrase (CA_ext_) activity by 4,4′–diisothiocyanatostilbene–2,2′–disulfonate (DIDS) and acetazolamide (AZ), respectively (= DIDS-AZ), and the reverse order of inhibition: the CA_ext_ activity first, followed by the AE port (= AZ-DIDS). Photosynthesis was measured under pH_T_ 9. Errors bars, ± SD, *n* = 3. *, *P *<* *0.01.

Under pH_T_ 9.0, initial blocking of the direct 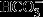 uptake through the AE port by DIDS resulted in 44% inhibition of the net photosynthetic rate (NPS) in *U. rigida*. Subsequent inhibition of the CA_ext_ by AZ to arrest the catalyzed dehydration of 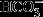 to CO_2_ resulted in an additional 39% inhibition of photosynthesis. When the order of inhibition for 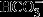 use was reversed, AZ and DIDS caused 29% and 26% of the NPS, respectively. Total inhibition of the NPS was higher when direct 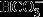 uptake through the AE port was blocked first (*T*-test, *P *=* *0.0195; Fig.2). Depending on the order of application of the inhibitors, 17–44% of NPS remained unaccounted.

In the absence of sequence information from *U. rigida*, the search for putative CCM elements in the EST libraries of *U. prolifera* (Jia et al. 2011) identified two *α*-CA and three *γ*-CAs (Table S1). However, the putative targeting signal peptides could not be resolved because the sequences retrieved from the EST data were not full length. Moreover, the exact localization of these proteins (e.g. chloroplast or periplasm) could not be defined. No *β*-CA was found in the EST libraries of *U. prolifera*. Furthermore, four putative HLA3 ABC transporters and three chloroplast carrier proteins (CCP1) and five mitochondrial transporter protein were found (Table S1). The transcriptome of a second species *Ulva linza* also identified putative CCM genes (Zhang et al. 2012). Among those found are at least one *α*-CA localized in the chloroplast lumen, several low CO_2_-induced proteins, a chloroplast Ci transporter (LCIB), several ABC transporters as well as the nuclear transcriptional regulators of CCM elements (CIA5, LCR1).

### Growth rate

**Figure 3:**
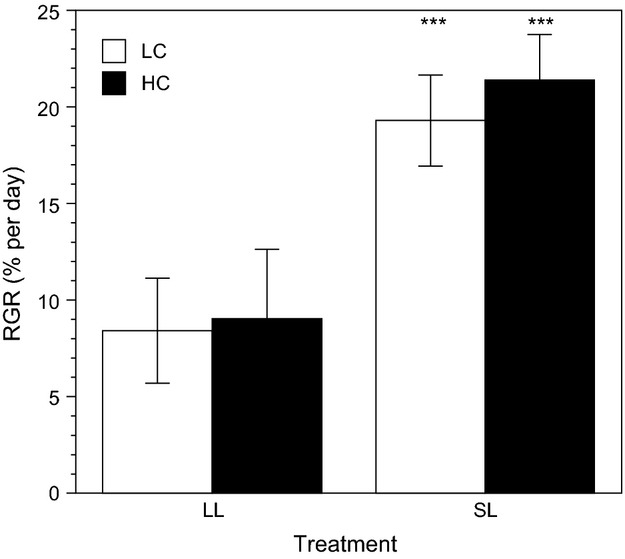
Relative growth rates (RGR) of *Ulva rigida* grown for 7 days under low and high *p*CO_2_ (LC = 471 *μ*atm and HC 1224 *μ*atm, respectively) and limiting and saturating light (LL = 31 *μ*mol photons m^−2^ s^−1^ and SL = 274 *μ*mol photons m^−2^ s^−1^, respectively). Errors bars, ± SD, *n* = 6. ***, *P *<* *0.001.

Relative growth rates (RGR; Fig.3) of *U. rigida* discs grown under limiting light (LL) were similar under low *p*CO_2_ (LC; 8.5 ± 2.7% d^−1^) and high *p*CO_2_ (HC; 9.1 ± 3.6% d^−1^). When discs were exposed to a saturating light (SL), RGRs were 2.27×  and 2.35×  higher under LC and HC, respectively (Fig.3). Statistical analysis showed that RGRs were strongly influenced by the experimental irradiance (ANOVA, *P *<* *0.001) but not by *p*CO_2_ (ANOVA, *P *=* *0.2473). The interaction between irradiance and *p*CO_2_ did not significantly affect the growth rate (ANOVA, *P *=* *0.5245).

### Chlorophyll *a* fluorescence

The maximum quantum yield of PSII (*F*_v_/*F*_m_) of *U. rigida* ranged from 0.807 to 0.813 (Table2). Neither light (ANOVA, *P *=* *0.152) nor the seawater carbonate chemistry (ANOVA, *P *=* *0.391) or their interaction (ANOVA, *P *=* *0.593) had an effect on *F*_v_/*F*_m_.

**Table 2:** Photophysiological parameters and tissue stoichiometry in *Ulva rigida* after seven-day incubation under different *p*CO_2_ and irradiance

Parameter	LL 31 *μ*mol photons m^−2^ s^−1^		SL 274 *μ*mol photons m^−2^ s^−1^	
	LC 471 *μ*atm pH_T_ 7.97	HC 1224 *μ*atm pH_T_ 7.59	LC 471 *μ*atm pH_T_ 7.97	HC 1224 *μ*atm pH_T_ 7.59
*F*_v_/*F*_m_ (rel. units)	0.813 ± 0.011	0.821 ± 0.008	0.807 ± 0.010	0.809 ± 0.023
ETR_max_ (*μ*mol electrons m^−2^ s^−1^)	28.9 ± 3.0	30.6 ± 5.6	42.2 ± 1.6	42.8 ± 3.5
*E*_k_ (*μ*mol photons m^−2^ s^−1^)	165.5 ± 16.9	169.6 ± 40.6	241.5 ± 9.1	245.3 ± 19.8
*α* (*μ*mol electrons *μ*mol^−1^ photons)	0.227 ± 0.008	0.227 ± 0.005	0.195 ± 0.012	0.187 ± 0.014
Chl*a* (*μ*g mg^−1^ FW)	1.45 ± 0.27	1.68 ± 0.12	1.09 ± 0.19	0.92 ± 0.21
Chl*b* (*μ*g mg^−1^ FW)	0.88 ± 0.13	0.95 ± 0.05	0.49 ± 0.05	0.42 ± 0.06
Chl*a*/*b*	1.64 ± 0.09	1.76 ± 0.14	2.19 ± 0.22	2.20 ± 0.30
C:N	9.7 ± 0.5	9.6 ± 0.3	10.5 ± 0.7	10.0 ± 0.8

Maximum quantum yield of PSII (*F*_v_/*F*_m_), electron transport rate-irradiance (ETR-*E*) curve parameters, that is, ETR_max_, *E*_*k*_, and *α* estimated using the hyperbolic tangent model of Jassby and Platt (1976), pigments, and molar C:N of *U. rigida* under different light (LL and SL; limiting and saturating light, respectively) and *p*CO_2_ concentrations (LC and HC; low and high *p*CO_2_, respectively). Data are means ± standard deviations (*n* = 6). FW – fresh weight.

All electron transport rate-irradiance (ETR-*E*) curve parameters (Table2), that is, ETR_max_, *E*_*k*_ and *α*, were significantly different between light (ANOVA, *P *<* *0.0001) but not significantly different between seawater carbonate chemistry (ANOVA, *P *>* *0.05). No significant interactive effect of the independent variables was observed in any of the ETR-*E* curve parameter (ANOVA, *P *>* *0.05). The ETR_max_ and *E*_*k*_ were significantly higher at saturating light (LL < SL; Tukey's HSD test, *P *<* *0.05), while the photosynthetic efficiency, *α*, was significantly higher at low light (LL > SL; Tukey's HSD test, *P *<* *0.05).

### Pigments

All LL-grown discs had a significantly higher biomass-based content of both Chl*a* and Chl*b* (Table2) compared with SL-grown discs (ANOVA, *P *<* *0.0001). The content of both chlorophylls was not affected by *p*CO_2_ (ANOVA, *P *>* *0.05). Light and *p*CO_2_ had an interactive effect on both Chl*a* (ANOVA, *P *=* *0.027) and Chl*b* (ANOVA, *P *=* *0.026). When grown under SL, the Chl*a* content was ∼35% lower than discs grown under LL. Similarly, the Chl*b* content in LL-grown discs was ∼50% higher than those grown in SL. The highest Chl*a*/*b* ratio of 2.2 (LC and HC) were calculated for SL-grown discs, whereas LL-grown discs had lower ratios of 1.64 (LC) and 1.76 (HC).

### Extracellular and intracellular carbonic anhydrase activity

**Figure 4:**
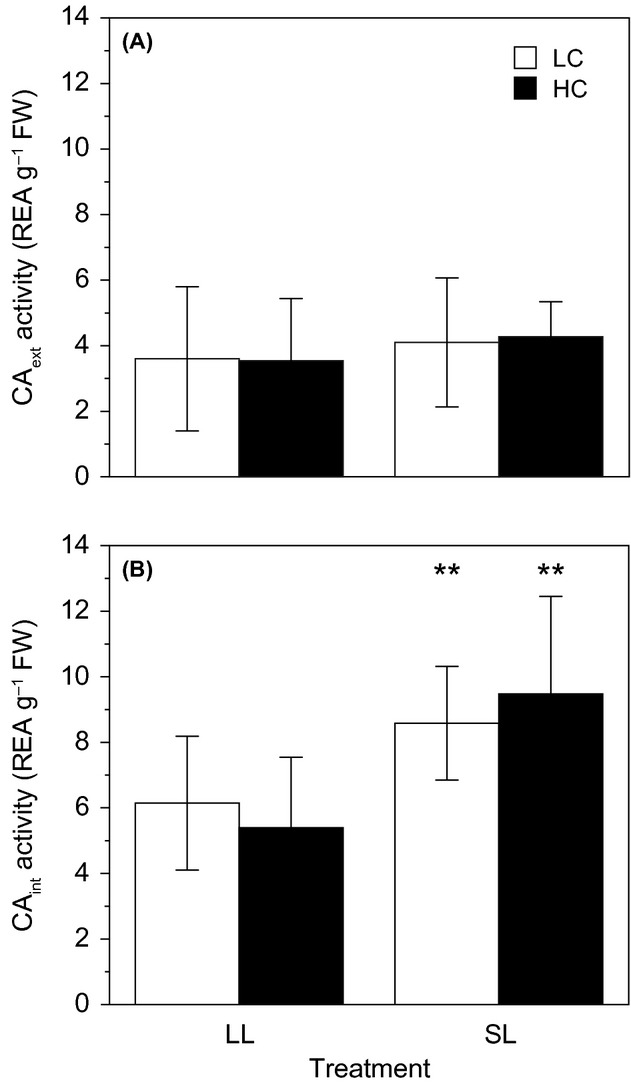
Relative enzyme activity (REA) of external (A) and internal (B) carbonic anhydrase activities in *Ulva rigida* after 7 days incubation under low and high *p*CO_2_ (LC = 471 *μ*atm and HC 1224 *μ*atm, respectively) and limiting and saturating light (LL = 31 *μ*mol photons m^−2^ s^−1^ and SL = 274 *μ*mol photons m^−2^ s^−1^, respectively). Errors bars, ± SD, *n* = 6. **, *P *<* *0.01.

The CA_ext_ activities (Fig.4A) ranged between 3.54 and 4.28 REA g^−1^ FW and were similar in all treatments (ANOVA, *P *>* *0.05), whereas CA_int_ activities (Fig.4B) were 20% higher under SL compared with LL (ANOVA, *P *=* *0.002) irrespective of *p*CO_2_. An increase in *p*CO_2_ (ANOVA, *P *=* *0.943) and the interaction between light and *p*CO_2_ (ANOVA, *P *=* *0.387) did not significantly change the CA_int_. Regardless of *p*CO_2_, the CA_int_ was 1.5–2× higher compared with the CA_ext_ under LL and SL, respectively.

### Carbon to nitrogen ratios and stable carbon isotope signatures

**Figure 5:**
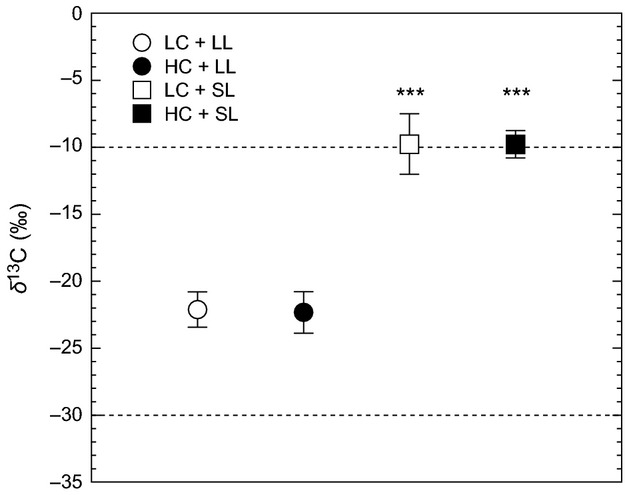
Stable carbon isotope signatures (*δ*^13^C) of *Ulva rigida* after seven-day incubation under low and high *p*CO_2_ (LC = 471 *μ*atm and HC 1224 *μ*atm, respectively) and limiting and saturating light (LL = 31 *μ*mol photons m^−2^ s^−1^ and SL = 274 *μ*mol photons m^−2^ s^−1^, respectively). Horizontal lines delimit the possible values for inorganic carbon use, that is, wholly dependent on CO_2_ (<−30‰) or 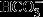 (>−10‰); values in between suggest mix use of CO_2_ and 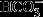 (*cf* Roleda and Hurd 2012). Errors bars, ± SD, *n* = 6. ***, *P *<* *0.001.

Molar C:N ratios (Table2) and *δ*^13^C signatures (Fig.5) of *U. rigida* differed significantly between light treatment, but there was no effect of seawater carbonate chemistry. Regardless of *p*CO_2_, the molar C:N ratio of LL-grown discs (LC = 9.7 ± 0.5; HC = 9.6 ± 0.3) was significantly lower than those in SL-grown discs (LC= 10.5 ± 0.7; HC = 10.0 ± 0.38; ANOVA, *P *=* *0.036; HSD test, *P *<* *0.05; LL < SL). Likewise, stable carbon isotope signatures (Fig.5) of LL-grown discs were similar between LC (–22.12 ± 1.31‰) and HC (–22.32 ± 1.53‰). When algal discs were grown under SL, these signatures shifted to a higher range under LC (–9.76 ± 2.24‰) and HC (–9.78 ± 1.01‰) (ANOVA, *P *<* *0.001; HSD test, *P *<* *0.05; LL < SL). The interaction between *p*CO_2_ and light did not significantly affect C:N ratio (ANOVA, *P *=* *0.517) nor the *δ*^13^C signatures (ANOVA, *P *=* *0.895).

## Discussion

Our study showed that 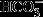 is the primary exogenous Ci source used by *Ulva rigida* to support growth and photosynthesis under saturating light, supporting the findings of previous work on other *Ulva* species (e.g. Björk et al. 1992; Drechsler et al. 1993; Larsson et al. 1997). However, when the known 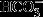 use mechanisms, that is, direct 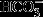 uptake through the AE port and external catalyzed dehydration of 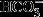, in *U*. *rigida* were inhibited, net photosynthesis (NPS) decreased by only 56–83% leaving the carbon uptake mechanism(s) for the remaining 17–44% of NPS unaccounted for. The remaining production cannot be attributed CO_2(aq)_ as the concentrations under pH_T_ 9.0 are much too low but rather to a possible light-dependent active 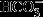 transport system. An in silico search of EST libraries of *U. prolifera* (Jia et al. 2011) found putative light-dependent 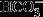 transporters, that is, the ABC transporters of the ABCC subfamily also found in *U. linza* (Zhang et al. 2012), which resemble the HLA3 transporter found in *Chlamydomonas* (Meyer and Griffiths 2013). The ABCC transporters are present in all eukaryotes and have different transport functions, including vacuolar sequestration of toxic metabolites and transport of chlorophyll catabolites during senescence, among others (Meyer and Griffiths 2013). The HLA3 analog has been implicated in the active bicarbonate uptake in *Chlamydomonas*: their role in a CCM is supported by genetic and physiological evidence (Wang et al. 2011).

Several CAs are involved in the utilization of 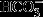. For example, the *α*-CAs, pCA1 and pCA2, catalyze the external dehydration of 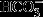 in *Chlamydomonas* (Spalding 1998); however, these isozymes are not found in *Ulva*. An unclassified CA_ext_ isozyme may be present in *Ulva,* but their contribution to the external dehydration of 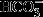 to CO_2_ and the active and/or passive transport of CO_2_ into the cell may not be sufficient to supply and fill the internal Ci pool to support the high growth rate of the species. Instead, two putative intracellular *α*-CA isozymes were found in the EST libraries of *U*. *prolifera* (Jia et al. 2011). These *α*-type CA isozymes also identified in *U. linza* (Zhang et al. 2012) most likely have a subcellular localization, for example, the chloroplast lumen. Both studies, that is, Zhang et al. (2012) and Ye et al. (2014), suggest that this *α*-CA isozyme is responsible for the internal 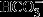 dehydration to provide the chloroplast with sufficient CO_2_ for carbon fixation. The same was recently reported in *Saccharina japonica*, where cDNA encoding *α*-CA was associated with the chloroplast envelopes and thylakoid membranes (Ye et al. 2014). Although the *β*-type CA was not found in the EST of *Ulva*, it is reported to be localized in the cytosol of another green alga *Coccomyxa* (Huang et al. 2011) where it facilitates Ci diffusion from the inner surface of the plasmalemma to the chloroplast envelope.

On the other hand, the exact roles of *γ*-type CA identified in the ESTs of *U*. *prolifera* are still unknown. They are usually associated with the mitochondria of green algae and plants (Parisi et al. 2004). Mitochondrial carbonic anhydrase (mtCA) is involved in enzymatic hydration of CO_2_, produced during respiration and photorespiration, to 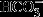 to stimulate anaplerotic *β*-carboxylation, where sufficient supply of 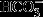 is required to support nonphotosynthetic biosynthetic pathways (Giordano et al. 2003). This may explain the nonphotosynthetic growth enhancement reported in *U. rigida* (Gordillo et al. 2001). This mechanism that recovers respiratory CO_2_ presents a very effective way to ensure efficient use of Ci for photosynthesis and nonphotosynthetic Ci-use pathways specially among calcifying algae and invertebrates (Roleda et al. 2012b).

The role of CA_ext_ in the Ci use of *U*. *rigida* was insignificant and was not regulated by either *p*CO_2_ or light. Despite the 160% increase in CO_2_ under the HC treatment (1224 *μ*atm), downregulation in CA_ext_ activity was not observed, a finding opposite to those found in the microalga *Emiliania huxleyi* (Richier et al. 2011). The constant low CA_ext_ activities (3.54–4.28 REA g^−1^ FW) across all treatments suggest that CO_2_ uptake after catalyzed external dehydration of 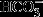 is not the main source of Ci in *U. rigida*. Considering that diffusive entry of aqueous CO_2_ into the cell is very slow in water, this mechanism is deemed to be insufficient for the accumulation of an internal Ci pool large enough to support the observed high growth rates (max 20% d^−1^) in this species. Moreover, CO_2_ uptake requires a conversion of CO_2_ to 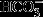 to maintain internal pH and avoid CO_2_ leakage from the cell (Price et al. 2008).

Conversely, CA_int_ was 1.5–2× higher under saturating light compared with limiting light. The higher enzymatic activity facilitates the conversion of 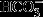 to CO_2_ to support the higher CO_2_ requirement of RuBisCO for photosynthetic fixation driving higher growth rate under saturating light. However, the source of this internal Ci pool (under high pH 9.0) cannot be wholly attributed to the known 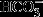 uptake mechanism where only up to 44% of net photosynthesis is supported by the direct 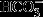 uptake through the AE port and 39% by the CA_ext_-mediated 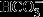 dehydration. The light-dependent HLA3 ABC transporters described above are hypothesized to most likely contribute to direct 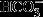 transport in order to saturate the internal Ci pool. 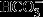 is the preferred Ci form for cellular accumulation because it is about 1000-fold less permeable to lipid membranes than CO_2_ (Price et al. 2008).

When active 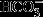 transport through the AE port was blocked first, inhibition of NPS during the 15-min period was 34% higher than when CA_ext_-mediated 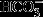 use was blocked first. Subsequent application of the second inhibitor contributed to additional 39% and 26% for the DIDS-AZ and AZ-DIDS treatment, respectively. In the AZ-DIDS treatment, where the CA_ext_-mediated 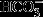 use was initially inhibited, algal discs were still actively transporting 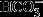 through the AE port that enables the cells to accumulate higher internal Ci pool contributing to lesser total inhibition of NPS. Conversely, when active 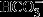 transport through the AE port was blocked first (DIDS-AZ), significantly higher total inhibition of NPS was observed. This suggests that different 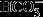 use mechanisms operate simultaneously and that the active transport through the AE port contributes more Ci to the internal pool.

Earlier studies of the carbon physiology of different *Ulva* species that focused on the AE port and external dehydration of 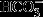 reported contradictory results. Larsson and Axelsson (1999) reported the net photosynthetic rates of different *Ulva* species are primarily supported by external dehydration of 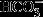 (34–68%) rather than direct 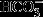 uptake (9–40%) through the AE port. Another study by Axelsson et al. (1999) reported >90% of *Ulva* *lactuca* photosynthesis is dependent on AZ-sensitive 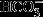 dehydration mechanism. Drechsler and Beer (1991) and Drechsler et al. (1993) reported a significantly higher contribution of direct 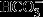 uptake compared with CA_ext_-catalyzed 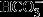 dehydration in *U. lactuca*. The above-mentioned studies on 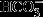 use mechanisms was measured between pH 8.2 and pH 8.7. At pH >9.4, only 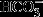 uptake via the putative DIDS-sensitive AE-transporter is operational in *Ulva* *intestinalis* (formerly *Enteromorpha intestinalis*) (Larsson et al. 1997). Conversely, only at extremely low pH 5.6 was a higher affinity for CO_2_ observed, which had no adverse effect on *U*. *lactuca*'s photosynthetic performance (Drechsler and Beer 1991). It should be noted that those *Ulva* species reported above may have some degree of taxonomical uncertainty. Previous physiological studies on *Ulva* did not identify that a light-dependent 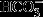 transporter is most likely operational in *Ulva*'s CCM as found in this study.

Elevated Ci (both CO_2_ and 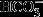) under OA did not cause a higher growth in *U. rigida* but growth rate was limited by light. This suggests that the present-day Ci concentration is already saturating for *Ulva*. Cyanobacterial ancestors of the green algae evolved effective 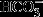 use mechanisms during the geologic low-CO_2_ environment (Giordano et al. 2005); we suggest that this trait is most likely genetically fixed and the modern *Ulva* can modulate their CCM under different *p*CO_2_ conditions (Giordano et al. 2005). Our results do not support the observation of Gordillo et al. (2001) on the same species and Xu and Gao (2012) on *U. prolifera* that higher *p*CO_2_ causes an increase in rates of both photosynthesis and growth. Although the response may be species specific, the mechanistic study of Gordillo et al. (2001) used extremely high CO_2_ in air (10 000 *μ*L L^−1^) and the identity of their *U*. *rigida* is uncertain as this was not molecularly identified (F. J. L. Gordillo, pers. comm.). The results of our study concur with those of Drechsler and Beer (1991) on *U. lactuca* where maximum photosynthetic O_2_ evolution was comparable under low (5.6) and high (8.2) pH. Furthermore, the study of Mercado et al. (2001) on the carbon physiology of three red seaweeds of the order Gelidiales inhabiting the intertidal zone also reported that photosynthesis is limited by light and not by Ci availability.

The seawater Ci species, that is, CO_2_ and 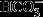, used as substrate for carbon uptake and fixation consist of stable carbon isotopes ^13^C and ^12^C within each species. The present seawater consist of 1% CO_2_ and 91% 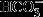; of all natural carbon, only 1.1% is ^13^C while 98.89% is ^12^C. Relative to ^13^*δ*_VPDB_ standard, CO_2(aq)_ has *δ*^13^C = –10‰ while dissolved 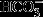 has *δ*^13^C = +1‰ to +1.5‰ (Mook et al. 1974; Mook 2005). The ^13^C/^12^C ratios (= *δ*^13^C) of the organic cellular material has been used as a proxy of Ci use relative to bulk seawater Ci source (Giordano et al. 2005). For example, organisms with *δ*^13^C higher than –10‰ (a value more positive than *δ*^13^C of CO_2_ in sea-water) must involve 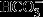 use (Raven et al. 2002). Among mixed CO_2_/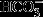 using algae, a significant use of CO_2_ under high *p*CO_2_ will shift *δ*^13^C signature corresponding to more CO_2_ use (i.e. toward –30‰) (Maberly et al. 1992). Here, we found that regardless of *p*CO_2_, *δ*^13^C signatures of *U*. *rigida* shifted upwards toward values of –10‰ and higher, under saturating light, which provides further support of the presence of a light-dependent active 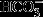 transport and intracellular accumulation, and the use of available 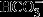 to support photosynthetic carbon fixation as previously suggested by Raven et al. (2002).

The C-isotope fractionation in aquatic plants is more complex than in terrestrial plants (Hoefs 2009). Factors that control the *δ*^13^C signature in algae include not only the availability of CO_2(aq)_, but also temperature, light intensity, nutrient availability, pH, and physiological factors such as cell size and growth rate (Hoefs 2009 and references therein). In *U*. *rigida*, the increase in productivity observed under saturating light regardless of increased availability of CO_2(aq)_ causes a corresponding rise in *δ*^13^C values, a response associated with more ^12^C locked up in the tissue as organic matter is generally depleted in ^13^C (Zeebe and Wolf-Gladrow 2001).

Moreover, the carbon fixation pathway can also influence the isotopic composition of organic matter. *δ*^13^C signatures between –32% and –22% are characteristics of C_3_ plants while *δ*^13^C between –16‰ and –10‰ are typical for C_4_ plants (Zeebe and Wolf-Gladrow 2001; Hoefs 2009 and references therein). The natural variations in the *δ*^13^C signature of *U. rigida* between –22‰ and –10‰ point to the possible occurrence of a C_4_ photosynthetic carbon fixation pathway, as observed in *U*. *linza* (Xu et al. 2013).

The rising atmospheric CO_2_ does not only trigger OA but also contributes to global warming that strengthens the vertical stratification of aquatic ecosystems: this suppresses the nutrient supply from deep water into the surface layer. The enhanced CO_2_ but reduced nutrient supply can therefore increase the C:N ratio of primary producers (e.g., phytoplankton) which are of low nutritional value to consumers (e.g., zooplankton), cascading throughout the entire aquatic food web (van de Waal et al. 2010). In our experiment, we increased CO_2_ by ∽160% and 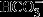 by ∽9% while the nutrient level remained constant. In this scenario, the enhanced exogenous Ci concentration and constant nutrient supply will theoretically still increase the C:N ratio. However, the insignificant fractional increase was observed relative to light and not to CO_2_ (Table2). This suggests that changes in the tissue/cellular stoichiometry in macroalgae may not be sensitive to a change in Ci alone. The small increase in C:N under saturating light suggests that exogenous Ci concentration is already saturating for *Ulva* regardless of *p*CO_2_ and saturating light increase carbon fixation.

The maximum quantum yield of PSII (*F*_v_/*F*_m_) and other photosynthetic parameters (ETR_max_, *E*_k_, *α*) are reliable proxies to assess seaweed photosynthetic performance under environmental stress such as high PAR, UVR, and temperature (e.g., Roleda et al. 2005; Rautenberger and Bischof 2006; Hanelt and Roleda 2009; Rautenberger et al. 2009; Roleda 2009). However, we suggest that these parameters are unlikely to be sensitive to changes in seawater carbonate chemistry, that is, *p*CO_2_ and H^+^, within the range likely to occur due to OA. In this study, rigorous PAM-based photophysiological measurements on *U*. *rigida* showed that ETR_max_, *E*_*k*_, and *α* are regulated by light, and a reduced seawater pH, simulating OA, had no effect, a finding contrary to that of Olischläger et al. (2013). Moreover, and contrary to the reported decrease in chlorophyll pigments under OA in *U*.* prolifera* (Xu and Gao 2012), OA did not affect the photosynthetic pigments of *U*.* rigida*: the amounts of Chl*a* and Chl*b* were regulated by light.

Algae regulate internal pH maintaining cytoplasmic pH at 7.3 ± 0.2 (Ritchie 1985; Lundberg et al. 1989; Smith and Bidwell 1989) which is 0.7 units lower than that of the current average surface seawater pH. Moreover, PSII is located in the thylakoid membrane where it is exposed to the acidified lumen (pH 5.0) and neutral to slightly basic stroma (pH 7.2–8.0) (Falkowski and Raven 2007). Therefore, the photosynthetic apparatus is already acclimated to a wide range of pH. The mechanism of how the bulk water pH may affect *F*_v_/*F*_m_ is unknown; studies reporting positive or negative effects of OA on this physiological proxy should therefore be interpreted with caution as they are possible artifacts.

In conclusion, *U*.* rigida* is insensitive to OA. The present-day seawater Ci pool is saturating for photosynthesis and growth, and these parameters were primarily controlled by light rather than elevated CO_2(aq)_. For photosynthetic carbon fixation, 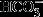 is the primary Ci species assimilated by *U*.* rigida*. Aside from the known catalyzed external 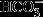 dehydration and direct 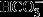 uptake through the AE port, another inhibitor-insensitive 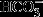 transport mechanism is most likely present. An in silico search of CCM elements in EST libraries of *Ulva* found putative light-dependent 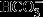 transporters, that is, the ABC transporters of the ABCC subfamily, found in both *U*. *prolifera* and *U. linza*. Neither a downregulation in extracellular CA-mediated 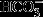 dehydration nor a shift to CO_2_ use was observed under high CO_2(aq)_. The shift in *δ*^13^C signatures in *U*. *rigida* toward –10‰ under saturating light under low and high CO_2(aq)_ but not toward –30‰ under elevated CO_2(aq)_ suggests preference and substantial internal 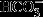 accumulation to support photosynthesis and growth regardless of CO_2_ concentrations. Despite the limited effect of OA and PPFR, the interaction of OA with other climate change stressors, for example, eutrophication and warming, may elicit different effects and warrants further investigation.

## Supporting Information

**Table S1.** Identification of putative carbon concentrating mechanism (CCM) elements from *Ulva prolifera* expressed sequence tag (EST) libraries (Jia et al. 2011).
