# Supplementary material for: Saturating light and not increased carbon dioxide under ocean acidification drives photosynthesis and growth in Ulva rigida (Chlorophyta)
Source: Ecol Evol. 2015 Jan 25;5(4):874–88. doi: 10.1002/ece3.1382 (PMC4338970; doi:10.1002/ece3.1382)
Supplement: Supplementary file 1 [file ece30005-0874-sd1.pdf]

SUPPORTING INFORMATION

Table S1: Identification of putative carbon concentrating mechanism (CCM) elements from *Ulva prolifera* expressed sequence tag (EST) libraries (Jia et al. 2011).

Chlamydomonas reinhardtii orthologue	*Ulva prolifera* EST accession number	Best blast hit NCBI NR database	E value (≤ 2E-8)
Alpha carbonic anhydrase (α-CA), CAH1 and CAH3	GW322697.1	α-CA, *Chlamydomonas reinhardtii*, AB65498.1	4E-10
	GW322456.1	Predicted protein with α-CA domain, *Chlorella vulgaris,* EFN59826.1	2E-8
Gamma carbonic anhydrase (γ-CA), CAG2	GW321210.1	Predicted protein (γ-CA domain), *Micromonas pusilla,* XP_003059787.1	5E-52
	GW324037.1	γ-CA, *Cicer arietinum*, XP_004512067.1	2E-39
	GW322710.1	Predicted protein (γ-CA domain), *Micromonas* sp., XP_002504597.1	9E-29
ABC transporter, HLA3	GW324213.1	Predicted protein (ABC ATPase superfamily domain), *Volvox carteri* f. *nagariensis*, XP_002945658.1	5E-75
	GW321231.1	Predicted protein (ABC ATPase superfamily domain), *Glycine max*, NP_001242603.1	8E-53
	GW324192.1	Predicted protein (ABC ATPase superfamily domain), *Glycine max*, NP_001242603.1	1E-50
	GW324904.1	Predicted protein (ABC ATPase superfamily domain), *Glycine max*, NP_001242603.1	3E-26
Chloroplast carrier protein, CCP1	GW320442.1	ATP/ADP translocator, *Chlorella vulgaris*, EFN54090.1	1E-105
	GW321159.1	ATP/ADP translocator, *Chlorella vulgaris*, EFN54090.1	4E-97
	GW321860.1	ATP/ADP translocator, *Chlorella vulgaris*, EFN54090.1	8E-91
Mitochondrial translocator	GW324803.1	Mitochondrial phosphate transporter, *Lotus japonicus*	4E-71
Mitochondrial carrier proteins	GW322149.1	Mitochondrial substrate carrier, *Volvox carteri* f. *nagariensis*, XP_002945883.1	5E-87
	GW320559.1	Predicted protein (mitochondrial carrier protein domain), *Chlorella variabilis*, EFN59790.1	1E-55
	GW322745.1	Predicted protein (mitochondrial carrier protein domain), *Chlorella variabilis*, EFN56490.1	5E-33
	GW321526.1	Predicted protein (mitochondrial carrier protein domain), *Chlorella variabilis*, EFN55963.1	4E-15

All *Ulva prolifera* EST entries listed above contain vector sequences. NR – non-redundant; E-value – expect value.

Supporting information to: Rautenberger, R., Fernandez, P., Strittmatter, M., Heesch, S., Cornwall, C. E., Hurd, C. L., and M. Y. Roleda*(2015): Saturating light and not increased carbon dioxide under ocean acidification drives photosynthesis and growth in *Ulva rigida* (Chlorophyta). *Ecology and Evolution*. *Corresponding author: Michael.Roleda@bioforsk.no, Tel.: +47 406 04 100.
